# Long-Term Safety and Efficacy of Prebiotic Enriched Infant Formula—A Randomized Controlled Trial

**DOI:** 10.3390/nu13041276

**Published:** 2021-04-13

**Authors:** Franka Neumer, Orenci Urraca, Joaquin Alonso, Jesús Palencia, Vicente Varea, Stephan Theis, Maria Rodriguez-Palmero, José Antonio Moreno-Muñoz, Francisco Guarner, Gigi Veereman, Yvan Vandenplas, Cristina Campoy

**Affiliations:** 1 BENEO-Institute, c/o BENEO GmbH, Wormser Str. 11, 67283 Obrigheim, Germany; stephan.theis@beneo.com; 2 Hospital de Nens, Consell de Cent 437, 08009 Barcelona, Spain; ourraca23@gmail.com; 3 CAP Llefià, Carretera Antiga de Valencia, s/n, 08913 Badalona, Spain; Joaquinalonso@terra.es; 4 Equipo Pediátrico San Francisco, Paseo de la Habana 27, 28036 Madrid, Spain; palenciagarrido@gmail.com; 5 Department of Gastroenterology, Hospital Sant Joan de Déu, 08017 Barcelona, Spain; vicentevarea@hsjdbcn.org; 6 Laboratorios Ordesa, Scientific Department, Parc Científic Barcelona, Baldiri i Reixac 15-21, 08830 Barcelona, Spain; Maria.Rodriguez@ordesa.es (M.R.-P.); JoseA.Moreno@ordesa.es (J.A.M.-M.); 7 Digestive System Research Unit, University Hospital Vall d’Hebron, 08035 Barcelona, Spain; fguarner@telefonica.net; 8 KidZ Health Castle, UZ Brussel, Vrije Universiteit Brussel, 1090 Brussels, Belgium; gveereman@skynet.be (G.V.); Yvan.Vandenplas@uzbrussel.be (Y.V.); 9 Centre of Excellence for Pediatric Research EURISTIKOS, Department of Pediatrics, University of Granada, 18071 Granada, Spain; ccampoy@ugr.es

**Keywords:** inulin, oligofructose, prebiotic, infant formula, safety, efficacy, infection

## Abstract

The present study aims to evaluate the effects of an infant formula supplemented with a mixture of prebiotic short and long chain inulin-type oligosaccharides on health outcomes, safety and tolerance, as well as on fecal microbiota composition during the first year of life. In a prospective, multicenter, randomized, double-blind study, *n* = 160 healthy term infants under 4 months of age were randomized to receive either an infant formula enriched with 0.8 g/dL of Orafti^®^Synergy1 or an unsupplemented control formula until the age of 12 months. Growth, fever (>38 °C) and infections were regularly followed up by a pediatrician. Digestive symptoms, stool consistency as well as crying and sleeping patterns were recorded during one week each study month. Fecal microbiota and immunological biomarkers were determined from a subgroup of infants after 2, 6 and 12 months of life. The intention to treat (ITT) population consisted of *n* = 149 infants. Both formulae were well tolerated. Mean duration of infections was significantly lower in the prebiotic fed infants (*p* < 0.05). The prebiotic group showed higher *Bifidobacterium* counts at month 6 (*p* = 0.006), and higher proportions of *Bifidobacterium* in relation to total bacteria at month 2 and 6 (*p* = 0.042 and *p* = 0.013, respectively). Stools of infants receiving the prebiotic formula were softer (*p* < 0.05). Orafti^®^Synergy1 tended to beneficially impact total daily amount of crying (*p* = 0.0594). Supplementation with inulin-type prebiotic oligosaccharides during the first year of life beneficially modulates the infant gut microbiota towards higher *Bifidobacterium* levels at the first 6 months of life, and is associated with reduced duration of infections.

## 1. Introduction

There is increasing evidence that colonization of the gut after birth has a long term “programming” effect on health as mediated through the immune system. This has led to the hypothesis that the gut microbiota has an important role in the modulation of the immune system, and therefore, in the prevention of infections in the infant. Feeding type is one of the main factors to influence this colonization process [1]. It is well-known that infants who have been breast-fed harbor bifidobacteria as predominant microorganisms and slower development of the gut microbiota structural diversity, while those who have been fed a standard formula develop a more varied microbiota with higher proportions of, e.g., *Escherichia coli, Clostridium* and *Bacteroides* (e.g., [2]).The bifidobacteria-stimulating effect has been ascribed to the presence of human milk oligosaccharides (HMO), which are the third-most prevalent component in mother’s milk [3]. These non-digestible carbohydrates have prebiotic effects by stimulating the growth of bifidobacteria in the infant gut microbial community and leading to the production of lactate and short-chain fatty acids (SCFA), in particular acetate, propionate and butyrate. SCFA have been shown to influence many molecular and cellular processes, linked to immunity and other physiologically relevant aspects [4,5,6]. Moreover, the gut microbiota, including its fermentation activity, has been considered as the most important and an early stimulus for the development of the gut-associated lymphoid tissue (GALT) [7]. Consequently, the establishment of gut microbiota is generally accepted as being of great importance to gastrointestinal physiology and to modulating the health and well-being of the infant [8].

When breast feeding is not possible, one of the proposed strategies for modulating the composition of the intestinal microbiota of the formula-fed infant and bringing it closer to breast-fed babies is the enrichment of infant formula with established prebiotics such as inulin-type fructans, such as oligofructose and inulin, and galacto-oligosaccharides (GOS) [9]. Similar to HMO, the fermentation of prebiotic carbohydrates leads to growth stimulation of bifidobacteria and the production of lactate and SCFA [10]. Two previous studies in healthy term newborns have demonstrated that infant formula supplemented with Orafti^®^Synergy1, a chicory-derived inulin which is enriched by shorter chain inulin (oligofructose), modulates the gut microbiota composition as well as stool characteristics closer and more comparable to those of breast-fed infants [11,12]. Prebiotics have also been found effective in decreasing the rate of infections and in reducing febrile episodes in infants and young children [13,14,15,16,17,18]. Furthermore, inulin and oligofructose can modulate certain functions of the immune system. In animal studies, inulin and oligofructose have been shown to affect different parameters and functions of the gut-associated lymphoid tissue (GALT), such as interleukin 10, the concentration of secretory IgA and the cytotoxicity of natural killer cells [19].

The aim of the present study was, therefore, to evaluate the effects of long-term supplementation of an infant formula with a mixture of short and long chain inulin-type oligosaccharides (Orafti^®^Synergy1) on immunological health-related outcomes, as well as on gastrointestinal function and well-being in infants during the first year of life. The effects on infant growth, tolerance and on the composition of fecal microbiota were further aims of this study. The hypotheses to be tested in the present study were: the administration of an infant formula and follow-on formula enriched with prebiotic oligosaccharides (Orafti^®^Synergy1; Beneo-Orafti, Tienen, Belgium) in a quantity of 0.8 g/dL during the first year of life improves the immunological status and reduces the incidence of infections and days with fever, improves gastrointestinal function and increases infant well-being.

## 2. Materials and Methods

### 2.1. Study Design

This clinical study was conducted as a multicenter, prospective, randomized, double-blind, placebo-controlled study in healthy term infants <4 months of age. It was part of the EARNEST project (EARly Nutrition programming-long term follow up of Efficacy and Safety Trials and integrated epidemiological, genetic, animal, consumer and economic research), an EU-funded integrated project within the 6th Framework Programme (FOOD-CT-2005-007036).

### 2.2. Study Participants

Infants were recruited from different pediatric hospitals, primary care centers and pediatric medical clinics located in Spain (five sites) and Belgium (two sites) from June 2008 until end October 2010. The recruitment of infants was done according to the following inclusion criteria: healthy term infants born between 37–42 weeks of gestation, birthweight appropriate for gestational age (between percentiles 3 and 97), between 0–4 months of age and exclusively fed infant formula on enrolment into the study. The exclusion criteria were: parents not able to comply with the study follow up (according to physician criteria), any relevant disease that could affect infant feeding and growth, any condition related to the immune system (e.g., primary immunodeficiency) and presence of an infection during 7 days prior to study entry. Although it was not specifically mentioned that antibiotics could not be taken, this is implicit in this exclusion criterion. Infants fed an infant formula containing probiotics or prebiotics 15 days prior to study entry were excluded from participation.

**Figure 1:**
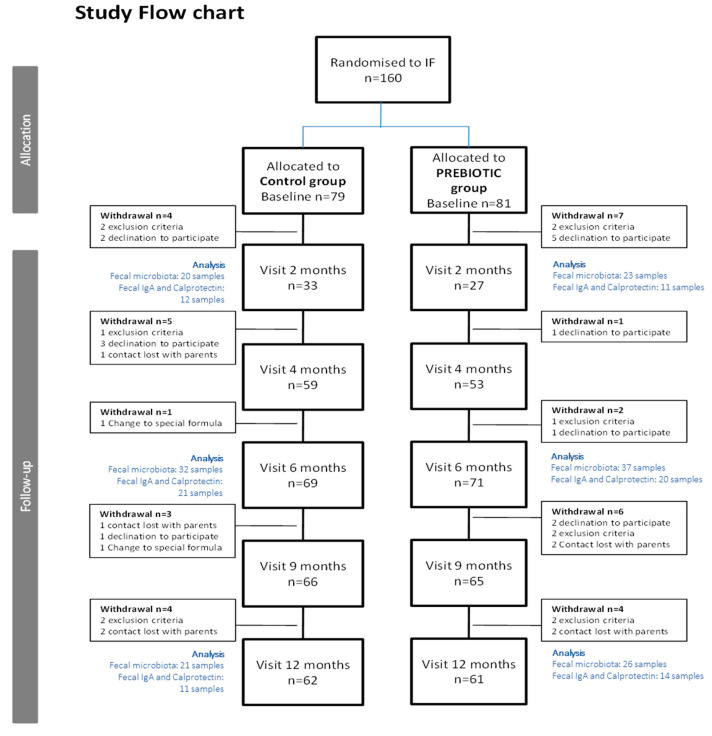
Flow chart of the infants included in the study (IF = Infant Formulae).

The total number of recruited infants was *n* = 160. Since infants between 0 and 4 months of age were included in the study, the data on study visit 2 (corresponding to 2-month-old infants) include a smaller number of children compared to later study visits. For more details, please see Figure 1 (study flow chart).

### 2.3. Intervention

The nutritional intervention lasted from the day of inclusion until the infant reached one year of life. Infants were randomly assigned to receive either a standard formula (control formula) or an experimental formula supplemented with 0.8 g/100 mL of chicory-derived oligofructose-enriched inulin (Orafti^®^Synergy1; Beneo-Orafti, Tienen, Belgium). Orafti^®^Synergy1 is an oligofructose-inulin combination containing shorter chain inulin (oligofructose DP < 10) and longer chain inulin (inulin DP ≥ 10) in an approximate 50:50 ratio ± 10% each.

Both control and experimental formulae had similar energy, macro- and micronutrient contents and were in accordance with the EU legislation on infant and follow-on formula composition (Directive 2006/141/EC). Both infant and follow-on formulae were developed to be administered during the study (see formula nutrient composition in Supplementary Table S1). Th energy content was 66–67 and 68–69 kcal/100 mL, protein content was 1.4 and 1.8 g/100 mL and lipid content was 3.5 and 3.2 g/100 kcal in infant and follow-on formulae, respectively. Neither control nor experimental formulae were supplemented with arachidonic and/or docosahexaenoic acid because it was not mandatory at the time the study was performed. Formulae were manufactured by Laboratorios Ordesa (Barcelona, Spain). They were coded by the sponsor using a color code, and research staff, pediatricians and parents were blinded throughout all the study period.

### 2.4. Outcomes

Primary outcome of the study was the occurrence of infections in infants during the first year of life (measured through the presence of fever >38 °C and number and duration of infectious episodes). Secondary objectives included gastrointestinal function and well-being, measures of infant growth, intestinal microbiota composition and fermentation products, development of allergy in the infant and effects on immunology markers in feces.

### 2.5. Study Procedures

Study protocol was approved by the ethical committee in all participating centers. At the recruitment visit, infants <4 months of age were randomized to receive either an infant formula supplemented with 0.8 g/100 mL of oligofructose-enriched inulin (Orafti^®^Synergy1) or a standard formula with no prebiotics added. Allocation to a study group was performed according to a computer-generated randomization list, which was provided to a study member not involved in recruitment or study visits. Infant follow-up was carried out up to the age of 12 months. Study visits with pediatricians were scheduled at baseline, 2, 4, 6, 9 and 12 months of age of the infants. Exclusion criteria during the study were digestive intolerance to either of the two study formulae, voluntary withdrawal by the parents, feeding with a formula different from the study formulae for more than 10 days during the study or regular supply of prebiotics or probiotics through other complementary foods (e.g., biscuits, yogurt, infant cereals, etc.).

At baseline visit, demographic information and infant characteristics were collected by interview. Baseline data on stool frequency and consistency, digestive tolerance, sleeping habits and infant crying duration were collected by parental recall over the past week. In addition, at all study visits, infant anthropometry was recorded: weight of the naked infant was determined by calibrated electronic scales, length was measured in supine position by using a standard measuring board and head circumference was determined using a non-extendable insertion tape. For anthropometric measurements, Z-scores were calculated according to the World Health Organization (WHO) standards using WHO software (WHO Anthro software). Furthermore, physical and clinical exploration was performed and variables related to lifestyle habits were recorded at each study visit.

Throughout the study, parents were advised to contact the pediatrician in case of any infectious or relevant event. In addition, they were given a diary to register data related to eventual infectious events: number of days with fever defined as infant body temperature >38 °C (thermometer was provided by the sponsor to all study participants and instructions to measure temperature were given to the parents), medical diagnosed infection and duration of the infection, as well as use of antibiotics or other medication. The parents’ diaries also included data on digestive tolerance (number of vomiting and regurgitation episodes per day, presence of excessive flatulence and presence of infantile colic), number and type of depositions per day, sleeping habits (total hours slept, total hours of nocturnal sleeping per day) and a crying diary, which were collected during the first week each study month throughout all the study period. Parents were asked to bring the diaries to each study visit in order to report on all the collected data to the study pediatrician.

Stool consistency was evaluated on a 1-to-3 scale with pictograms (1 = hard, 2 = soft/formed, 3 = liquid/semi-liquid). For further analysis, a reciprocal stool consistency score was done and calculated as follows:

(1) $$Score=\frac{\left(Numbertype1\times 3)+(Numbertype2\times 2)+(Numbertype3\times 1\right)}{Totalnumber}$$

The lower score values reflect softer stools, while higher ones reflect harder stools.

From the 5th month of age, instructions to start complementary feeding were provided by the pediatrician, following guidelines included in the study protocol, which were based on national recommendations. At each study visit, feeding data were collected (total volume of study formula ingested per day, introduction of complementary foods). Any potential adverse event (AE), defined as any untoward medical occurrence in an infant recruited in the study which does not necessarily have a causal relationship with the intervention with study formula was also recorded.

### 2.6. Analysis of Fecal Microbiota

Stool samples were collected from a subgroup of infants at the age of 2, 6 and 12 months, in four study centers which had equipment needed to store samples. After providing materials for sample collection in the previous visit, around 1–2 mL of feces obtained by spontaneous defecation were collected in a sterile plastic container, frozen as soon as possible and kept in a freezer at −20 °C. Stool samples were brought frozen to the study unit at specific study times. Stool samples were stored at the hospitals at −80 °C and then sent frozen to a central laboratory (Institut für Mikroökologie, Auf den Lüppen, Herborn, Germany). Stool samples were analyzed for *Bifidobacterium, Bacteroides, Enterobacteriaceae, C. coccoides* group, *C. leptum* group and total bacteria by qPCR with the procedure described below. Different protocols and primer pairs were applied for the detection of the individual bacteria [20,21,22] (Supplementary Table S2).

From the stool samples, DNA was extracted using Easy Mag DNA Isolation system (BioMerieux, Nuertingen, Germany) according to the manufacturer’s instructions. PCR amplification and detection was performed using an ABI 7300 Real Time PCR System (Applied Biosystems, Darmstadt, Germany) in optical-grade 96-well plates sealed with the optical sealing tape. Each reaction mixture (25 µL) comprised 12.5 µL of QuantiTect SYBR Green PCR Master Mix (Qiagen, Hilden, Germany), 1.6 µL of primer mixes (10 mol/µL each), 9.4 µL of sterile distilled water and 1.5 µL of stool DNA (10 ng/µL). For the negative control, 1.5 µL of sterile distilled water instead of the template DNA solution was added to the reaction solution. A standard curve was produced using the appropriate reference organism to quantify the qPCR values into numbers of bacteria per gram of stool. Each reference species was grown to sufficient amounts in the appropriate media and under the required conditions (for further information please check www.dsmz.de, accessed on: 14 April 2011). The exact cell numbers were determined by flow cytometry. Subsequently, the highest attained cell numbers were diluted in 10-fold steps and the DNA was extracted. Following qPCR, the Ct for each dilution was determined and plotted on a curve. This curve determined the standard curve for each reference organism. The following species were used as reference organisms for the respective standards: *Bifidobacterium adolscentis* DSM20083 for the genus *Bifidobacterium*, *Bacteroides fragilis* DSM 1396 for the genus *Bacteroides*, *Escherichia coli* DSM 301 for the family *Enterobacteriacae*, *Blautia coccoides* DSM 935 for the *C. coccoides* group and *Clostridium leptum* DSM 753 for the *Clostridium leptum* group. DNA from the reference species was isolated with the same protocol as described earlier. The standard curves were prepared using the same PCR assays as for the samples. The fluorescent products were detected in the last step of each cycle. A melting curve analysis was carried out after amplification to distinguish the targeted PCR products from the non-targeted PCR products. The melting curves were obtained by slow heating at temperatures of 60 °C to 95° C at a rate of 0.2 °C/s, with continuous fluorescence collection. The data was analyzed using the ABI 7300 Real Time PCR System Sequence Detection Software Version 1.4

The real-time PCRs were performed in triplicate, and average values were used for enumeration. The amplification program used consisted of one cycle of 95° C for 15 min, 40 cycles of 95 °C for 30 s and 50 cycles of 60 °C for 60 s (depending on primers, see Supplementary Table S2 annealing temperatures). The detection limit was 10^5^ cfu/g of wet feces.

### 2.7. Biochemical Analysis

Calprotectin was evaluated in fecal samples by calprotectin enzyme-linked immunosorbent assay (ELISA) (Calprest; Eurospital SpA, Trieste, Italy) according to the manufacturer’s instructions. Briefly, stool samples were thawed and 40–120 mg suspended in an extraction solution in a weight per volume ratio of 1:50, mixed by vortex and homogenized. One milliliter of the homogenate was centrifuged for 20 min at 10.000 g and supernatants were frozen at −20 °C until analysis. After at least 1:50 dilution in the buffer, supernatants were added to the microtiter plate wells, incubated at room temperature for 45 min and washed three times with washing solution. Purified rabbit anti-human calprotectin antibodies, conjugated with alkaline phosphatase, were added and incubated for 45 min at room temperature. After washing, enzyme substrate was added to each well and 30 min later, the reaction was stopped with 1 M NaOH. Optical densities were read at 405 nm with a microplate ELISA reader (Multiskan EX; Thermo Electron Corporation, Helsinki, Finland). Samples were tested in duplicate and results were calculated from standard curve and expressed as μg/g feces. Secretory IgA was analyzed from the supernatants of fecal homogenates. Briefly, the homogenates were centrifuged (13.000× *g* per 10 min), and the supernatants were collected. sIgA was determined by indirect enzyme immunoassay (Salimetrics LLC, Carlsbad, CA, USA). The results were expressed as µg/g of feces.

The study was performed following the Helsinki declaration and the guidelines for the ethical conduct of medical research involving children. Study protocol was approved by Ethical Committees in all participating hospitals and the CEIC Fundacio Sant Joan de Déu (Central Ethical Committee Spain, approval No: 2008). Written informed consent was obtained from parents or tutors of the infants. The trial has been registered at ClinicalTrials.gov (identifier NCT04441359).

### 2.8. Statistical Analysis

Sample size calculation was performed based on potential differences in the variable days of fever between intervention groups. The estimates were a difference in 0.66 days of fever (1.3 common standard deviation) between the control and experimental group [23], an α error of 0.05, a β error of 0.2 and a power of 80%. Assuming a 20% attrition rate, a total of 154 infants, 77 infants in each group, were required.

Once study results were available, a statistical database was developed in the Macro system. Complete data from infants both from case report forms and parent’s diaries were introduced in the database. For all diary data, a daily average or a daily total was calculated for those parameters where more than one entry per day was possible (e.g., number of stools per day). These data were later assigned to the corresponding study visits. For variables referring to infants’ well-being, such as total sleeping time, nocturnal sleeping time (from 22 h to 10 h) and crying time, we obtained half-hour fractions. We added all the amounts for each week, transformed values to minutes/24 h, and assigned diary data to the corresponding study visits. Once all the data were introduced and reviewed for queries, they were exported and listed. After doing blinding review of variables listed, the study population was defined.

Data of the continuous variables are presented as the mean ± SD and those of categorical ones as counts and percentages. Normality of the data was evaluated by the Kolmogorov-Smirnov test. Comparisons between groups for demographical variables over visits were done fitting a linear mixed model with time and group as the main effects.

The statistical analysis of outcome variables with respect to the a priori hypothesis of the study was conducted as follows: the total number of days with fever and total number of infections were modeled fitting a generalized linear model of Poisson with the treatment group as the independent variable. Marginal distributions of the number of days with events or number of infections along time were described fitting a Poisson model using a generalized estimating equation approach (GEE) with length of the time during which the events are recorded and treatment group as independent variables. Cumulated days of fever were compared between treatments using two sample median tests. For binary outcomes repeated over time, the GEE approach assumed a binomial distribution, the logit of event being explained by time, treatment group and their interaction. IgA and calprotectin were transformed to log in order to get centered distributions. Comparisons between groups for IgA and calprotectin were done fitting a linear mixed model with time and group as main effects. Stool consistency scores were compared between groups fitting a rank analysis of covariance, with comparisons made at 2, 4, 6, 9 and 12 months, taking the baseline value as a covariate. Final and baseline values were ranked and a linear regression of final value ranks on baseline ranks was fitted to obtain residuals. These mean values of residuals in both treatment groups were compared by means of the Mantel-Haenszel mean score statistic.

Data management and statistical analysis was performed with SAS 9.4 (2002–2010 by SAS Institute Inc., Cary, NC, USA). The microbiota data assessed by qPCR are presented as absolute cell counts (log transformed) and as the percentage of the total bacteria (calculated with non-transformed data). Several samples were below the detection limit, which was 5 log cfu/g feces. To account for the missing values, for the statistical evaluation, the missing values were imputed by the detection limit of 5 log. All groups and assessment time points were treated similarly. The Kolmogorov-Smirnov test has been performed to test the normal distribution of absolute cell counts. The median (interquartile ranges, IQR) is shown. The Mann-Whitney test was used for a comparison of the two groups at month 2, 6 and 12.

## 3. Results

The formula enriched with Orafti^®^Synergy1 was safe and well-tolerated by the infants. The rate of serious adverse events was low (*n* = 1 infant in prebiotic group had to be hospitalized for an event that was not related to formula intake, estimated by a physician).

In total, *n* = 160 infants were recruited from different clinical centers in Spain and Belgium. The ITT (intention to treat) population consisted of 149 infants, and the PP (per protocol) population of 123 infants (a study flow chart is presented in Figure 1). The baseline characteristics of the enrolled infants are presented in Table 1. There were no statistically significant differences between the groups.

### 3.1. Anthropometric Data

**Figure 2:**
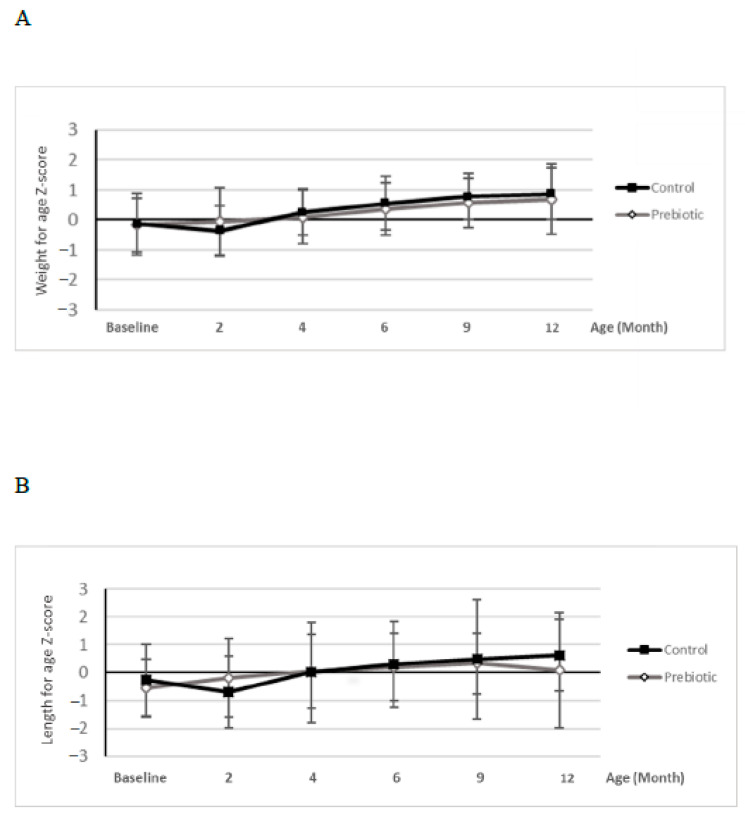
Mean data for weight–for–age (**A**) and length–for–age (**B**) WHO growth standard Z–scores for prebiotic and control groups during the course of the study (until 12 months of age). Grey lines containing white rhombs represent the prebiotic group, while black lines with black squares represent the control group. Lines represent Standard Deviation (SD). There were no group-related differences.

There were no significant differences in growth-related variables measured as Z-scores (weight-for-age, length-for-age) between the two feeding groups (Figure 2). Supplementary Table S3 presents the results on body weight (g), length (cm) and head circumference (cm). Average formula intake (mL/day) was in the normal range [11] in both groups at all study points, yet with a significantly higher average intake with the standard formula at month 4 (Supplementary Table S4).

### 3.2. Health-Related Outcomes

**Figure 3:**
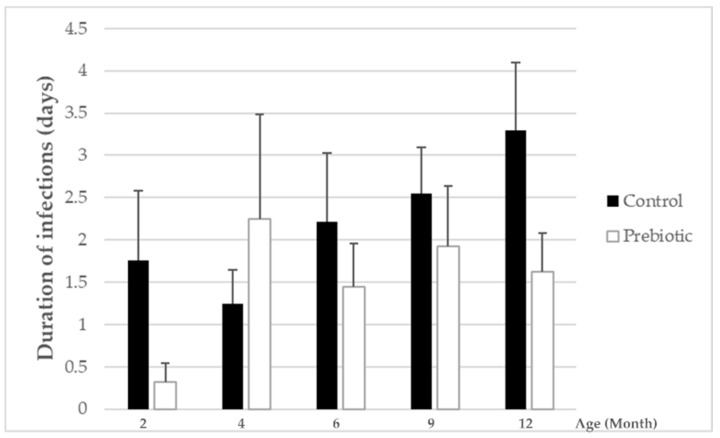
Mean duration of infections between the prebiotic (white bars) and control group (black bars) for months 2, 4, 6, 9 and 12. Lines represent the SEM (Standard Error of the Mean). Statistical difference between groups for total mean duration of infections was analyzed via the generalized estimating equation (GEE) approach (significantly lower with prebiotic group, *p* = 0.034).

The total number of infections was low and did not differ between the two groups (Supplementary Table S5), whereas the mean duration of infection was significantly lower in the group receiving the prebiotic-supplemented formula (intervention group) compared to the standard infant formula group (control) (*p* = 0.034, Figure 3). The number of infants who experienced at least one infection was slightly lower in the intervention group compared to the standard infant formula group, without reaching statistical significance (51 vs. 62%, respectively, *p* = 0.186). The cumulated days with fever at 12 months were small and comparable between the two feeding groups (2.44 ± 2.27 d vs. 2.59 ± 2.59 d for the prebiotic and control group, respectively, *p* = 0.831).

The number of infants with exanthema, atopic disease and allergies was overall very low throughout the follow-up visits. Although there were no statistically significant differences between the groups for all of these three conditions (*p* > 0.05), fewer infants of the intervention group were affected at months 2, 6, 9 and 12 (cumulative data for exanthema: 1 in prebiotic vs. 3 in control group; dermatitis: 2 in prebiotic vs. 5 in control group; allergies: 0 in prebiotic vs. 1 in control group). With respect to stool biomarkers of inflammation, slightly higher levels of sIgA were found in the intervention group, particularly at 12 months of age (*p* = 0.078). Levels of fecal calprotectin did not differ between both groups (data not shown).

### 3.3. Infant Behaviour and Well-Being

**Figure 4:**
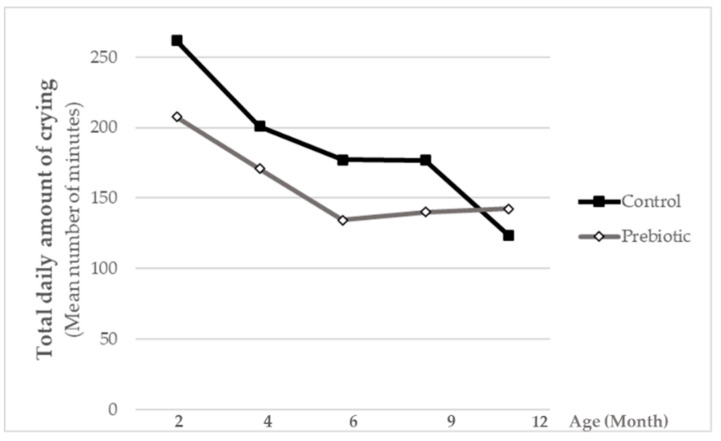
Total daily amount of crying presented as the mean number of minutes. Statistical differences between groups were analyzed via the generalized estimating equation (GEE) approach.

Total daily amount of crying was lower (*p* = 0.0594) in the intervention group compared to the control group for most of the studied period (i.e., in 2-, 4-, 6- and 9-month-old infants) except for the last time point (Figure 4).

The presence of digestive symptoms is shown in Supplementary Table S6. Measurements related to digestive tolerance (number of regurgitations/day, number of vomits/day, presence of flatulence or meteorism) were not different between prebiotic and control groups at all time points. Similarly, infantile colic and resting times were comparable between both groups (data not shown).

### 3.4. Stool Parameters

**Figure 5:**
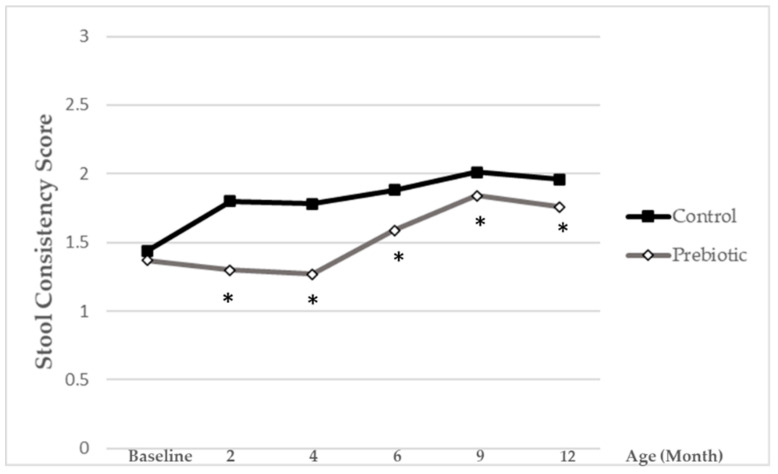
Mean stool consistency score for infants receiving prebiotic or control formula until 12 months of age. Grey lines containing white rhombs represent the prebiotic group, while black lines with black squares represent the control group. The lower score values reflect softer stools, while the higher ones reflect harder stools. * Statistically significant from control group (*p* < 0.05).

The total number of stools per day was comparable between both feeding regimes throughout the study (data not shown). Stools of infants receiving the formula supplemented with Orafti^®^Synergy1 scored lower (and were softer) than stools of infants in the control group fed a standard non-supplemented formula throughout all study period (*p* < 0.05) (Figure 5).

### 3.5. Fecal Microbiota Composition

**Figure 6:**
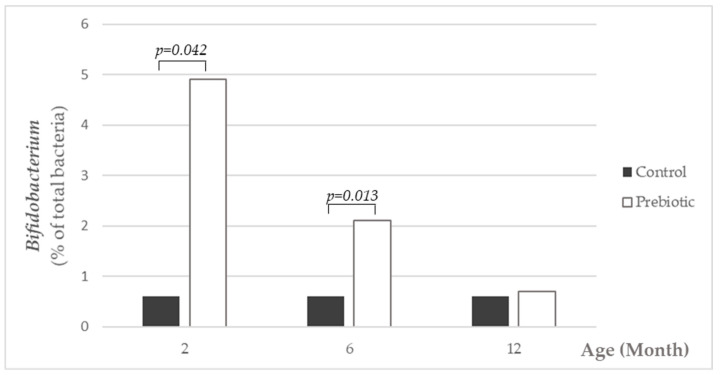
Fecal *Bifidobacterium* counts by group (median) of the percentage of total bacteria at months 2, 6 and 12. The black bars represent the control group, while the white bars indicate the prebiotic-supplemented group. *Bifidobacterium* counts were significantly higher in the prebiotic group at months 2 and 6 of age.

Total bacteria counts were similar in both groups at all time points (months 2, 6 and 12). *Bifidobacterium* counts were significantly higher in the prebiotic compared to the control group (8.91 (8.31, 9.41) vs. 8.15 (7.24, 9.09), median and IQR intervals, respectively) at 6 months of age (Table 2). When results were expressed as proportions of *Bifidobacterium* in relation to total bacteria, the prebiotic group showed significantly higher values at month 2 and 6, but not at month 12 (Figure 6). Bacterial counts and proportions of other bacteria groups did not differ between both groups.

## 4. Discussion

The addition of Orafti^®^Synergy1 to standard infant formula at a daily dose of 0.8 g/dL was well tolerated by the infants, and furthermore, slightly increased their well-being, as illustrated by numerically lower daily crying-times compared to the non-supplemented control.

The good tolerance of the supplemented formula was demonstrated by similar formula intake volumes and growth-related parameters in the two groups of infants throughout the study. Typical gastrointestinal symptoms (e.g., regurgitation, vomiting) did not differ between the groups. The rate of adverse events was low and comparable as well. No safety concerns were revealed as reflected by the absence of relevant differences in number, severity, relatedness or type of AEs. The present study thus confirms findings of previous studies reporting that Orafti^®^Synergy1, at the same dose level, is safe and well-tolerated by neonates [12] and newborn infants [11]. Overall, the results are in line with earlier clinical trials performed in healthy infants investigating safety and tolerability of formulae enriched with prebiotic inulin-type fructans (e.g., [24,25,26,27]). The current study also agrees with the conclusions of a recent systematic review on the safety of infant formulae supplemented with established prebiotics [8]. The study, nevertheless, is distinct from previous ones, as it is a particularly long intervention and covers the first 12 months of life.

The main objective of the present trial was to explore whether supplementation of prebiotic chicory-derived inulin-type fructans could reduce the incidence of infections in newborn infants until the age of 1 year. A recent systematic review and meta-analysis addressed the efficacy of prebiotics in the prevention of acute infectious diseases in the healthy pediatric population [15]. The authors concluded that prebiotics may be effective in decreasing the rate of infections requiring antibiotic intake, and in lowering the rate of overall infections in infants and children aged 0–24 months, while the number of studies investigating the preventive effects of prebiotics on acute infections is limited. Consequently, the current study provides further support for such health-related outcomes.

In general, the overall number of infections as well as days with fever were very low during the whole study period. This indicates that the infants included in this study were very healthy, a fact that may have hampered the possibility to see more clearer beneficial effects with prebiotic-supplemented vs. non supplemented formula on these outcome parameters. However, while the total numbers of infections were not influenced by prebiotic intake, the overall duration of infections was significantly lower in the group receiving the Orafti^®^Synergy1-enriched formula compared to the group receiving the standard formula. This reveals that the prebiotics included in the infant formula might have induced a modulation of some immunological processes, presumably as a result of the microbiota modifications and related gastrointestinal immunological parameters. These may include, e.g., secretory IgA, for which a trend for an increase in fecal samples of the prebiotic-supplemented formula group was observed. Higher fecal concentrations for sIgA have also been reported in infants receiving an infant formula enriched with another prebiotic mixture (GOS:FOS) (e.g., [28,29]) and suggested to reflect an increase of mucosal immunity. Further studies with this prebiotic mixture found a reduced number of episodes of infections including recurrent infections [16], and also observed a lower incidence of atopic dermatitis at the end of the observation period of 6 months [30]. Moreover, the cumulative incidence of atopic disease during the two-year follow-up period was significantly reduced [31]. In the present study, the number of infants with exanthema, atopic disease and allergies was very low throughout the follow-up visits. Nevertheless, a numerically lower incidence of these diseases was observed in the prebiotic-fed babies covering most of the entire follow-up period.

Ingestion of infant formula supplemented with a mixture of short and long chain inulin-type oligosaccharides increased *Bifidobacterium* populations in the fecal microbiota in infants in the current study, and thus, this study confirms previous studies [11,12]. The pronounced differences in gut microbiota composition between prebiotic-supplemented formula and non-supplemented formula observed at months 2 and at 6 months of age disappeared as the ingestion of total amounts of infant formula decreases and diversification of the infant’s diet occurs; this effect could also be attributed to the increase of gut microbiota diversity within the maturation process. However, this early microbiota modulation is associated with longer term beneficial effects on clinical health and well-being up to 1 year of age. These data support the idea that very early life nutritional interventions can modify gut microbiota establishment, and consequently, its composition and function being determinant for immune system development and immune responses later in life [32,33,34].

Stool consistency was softer with the prebiotic-supplemented formula compared to the standard formula. This is in accordance with previous studies reporting a stool consistency closer to that of breast-fed infants. Due to the prebiotic nature of Orafti^®^Synergy1, its fermentation can benefit bowel habits and stool characteristics in formula-fed infants, similar to HMO in breast-fed infants. Previous reviewed trials consistently demonstrated that the addition of prebiotics to infant formulae has the potential to soften stools (e.g., [11,17,24,35]), an effect beneficial in particular in infants with hard stools [8,36].

A strength of this project is that it is a long-term study where infants were followed up to the age of 1 year. The findings of good tolerance and safety of Orafti^®^Synergy1 oligofructose-enriched inulin shown in previous studies have been confirmed in this study as well (good growth, a similar rate of AEs in both feeding groups). Drop-out rates were quite low considering the long-term nature of this study, and evenly distributed among the groups (21% vs. 25%, control vs. prebiotic group, respectively). This suggests that the drop-outs were not related to the intervention.

A potential limitation of this study could be that recruitment of infants was allowed between 0 and 4 months of age, which may increase the influence of other types of feeding and breastfeeding. However, randomization should have limited the between-group differences, as confirmed by the mean age being similar in both feeding groups at recruitment. Another limitation may be that this study applied qPCR on individual bacterial groups only to characterize the fecal microbiota of the infants during the intervention period. This limited the possibilities of doing a more explorative analysis about the relations of microbiota characteristics and health outcomes or other factors. In order to comprehensively understand the effects of early life nutritional interventions with prebiotics and their beneficial effects, further studies are, thus, warranted, including 16S rRNA gene sequencing techniques and long-term follow-up.

In conclusion, supplementation of infant formula with inulin-type prebiotic oligosaccharides containing shorter and longer chains was well tolerated and beneficially modulated the infant gut microbiota towards higher *Bifidobacterium* levels, accompanied by softer stool consistency. The reduced duration of infections emphasizes the possible interaction between gut flora modulation and immunity.

## Figures and Tables

**Table 1:** Baseline characteristics of the infants enrolled in the study.

		Prebiotic Formula		Control Formula	
**Data at inclusion**					
			mean (SD)		
Age (days)		67.87	(39.73)	60.04	(41.35)
Weight (g)		5216	(1344)	5088	(1529)
Height (cm)		56.69	(4.82)	56.57	(5.68)
Head circumference (cm)		38.75	(2.82)	38.73	(2.99)
Duration of breastfeeding (days)		45.88	(34.41)	47.71	(36.04)
Amount of formula ingested (ml/day)		718.8	(212.6)	790.8	(294.4)
**Data at birth**					
			mean (SD)		
Weight (g)		2882	(1081)	3704	(1243)
Height (cm)		49.61	(2.89)	49.92	(1.84)
Head circumference (cm)		34.29	(1.61)	34.94	(1.22)
Gestational age (years)		39.27	(1.47)	39.05	(1.57)
Maternal age (years)		31.38	(4.96)	32.77	(5.83)
Type of birth					
	Eutocic (%)		61.5		43.1
	Dystocic (%)		15.4		20
	Cesarean (%)		23.1		36.9
Sex	Boy (%)		47.7		49.2
	Girl (%)		52.3		50.8
Siblings	Yes (%)		51.6		51.6
	No (%)		48.4		48.4

* Statistical differences between the groups (*p* < 0.05).

**Table 2:** Fecal microbiota counts by group (median (IQR) of log cfu/g feces and statistical comparison of prebiotic vs. control group at months 2, 6 and 12).

	Age (Month)	Prebiotic Formula Median (IQR) log cfu/g Feces	Control Formula Median (IQR) log cfu/g Feces	P Prebiotic vs. Control
n	M2	23	20	
	M6	37	32	
	M12	26	21	
*Bacteroides*	M2	5.79 (5.00, 7.58)	6.47 (5.12, 7.76)	0.513
	M6	7.77 (5.38, 9.32)	6.01 (5.00, 8.97)	0.158
	M12	9.28 (8.22, 9.87)	9.51 (8.53, 10.22)	0.374
*Bifidobacterium*	M2	8.52 (8.00, 9.08)	8.16 (7.41, 8.88)	0.342
	M6	8.91 (8.31, 9.41)	8.15 (7.24, 9.09)	0.006
	M12	8.54 (7.80, 9.34)	8.40 (7.52, 9.07)	0.521
*C. coccoides* group	M2	5.00 (5.00, 5.31)	5.00 (5.00, 6.31)	0.884
	M6	5.88 (5.00, 6.81)	6.14 (5.00, 7.28)	0.437
	M12	7.11 (6.02, 8.00)	7.14 (6.54, 7.89)	0.638
*C. leptum* group	M2	5.00 (5.00, 5.00)	5.00 (5.00, 5.00)	0.125
	M6	5.00 (5.00, 5.59)	5.11 (5.00, 6.29)	0.078
	M12	7.00 (6.20, 7.59)	6.48 (5.70, 7.69)	0.585
*Enterobacteriaceae*	M2	7.76 (7.03, 9.05)	8.19 (7.37, 9.14)	0.932
	M6	7.96 (7.20, 8.44)	8.05 (6.71, 8.93)	0.732
	M12	7.00 (5.75, 7.52)	7.11 (5.92, 8.18)	0.404
Total counts	M2	10.17 (9.97, 10.48)	10.93 (10.04, 11.45)	0.053
	M6	10.55 (9.82, 11.31)	10.63 (10.19, 11.16)	0.777
	M12	10.40 (9.96, 11.12)	10.40 (9.99, 11.03)	0.789

## Data Availability

The data presented in this study are available on request from the corresponding author. The data are not publicly available.

## Supplementary Materials

The following are available online at https://www.mdpi.com/article/10.3390/nu13041276/s1, Table S1: Study formula characteristics, Table S2: Primer selection for qPCR, Table S3: Growth-related variables of infants following the consumption of either prebiotic vs. control formula at the following time points: age of 2, 4, 6, 9 and 12 months, Table S4: Amount of formula ingested following the consumption of either prebiotic vs. control formula at the following time points: age of 2, 4, 6, 9 and 12 months, Table S5: Number of infections until 12 months of age, Table S6: Digestive symptoms of the infants by feeding group and study time.

## References

[1] Penders J., Thijs C., Vink C., Stelma F.F., Snijders B., Kummeling I., van den Brandt P.A., Stobberingh E.E. (2006). Factors influencing the composition of the intestinal microbiota in early infancy. Pediatrics.

[2] Harmsen H.J., Wildeboer-Veloo A.C., Raangs G.C., Wagendorp A.A., Klijn N., Bindels J.G., Welling G.W. (2000). Analysis of intestinal flora development in breast-fed and formula-fed infants by using molecular identification and detection methods. J. Pediatr. Gastroenterol. Nutr..

[3] Kunz C., Rudloff S. (1993). Biological functions of oligosaccharides in human milk. Acta Paediatr..

[4] Gibson G.R., Probert H.M., van Loo J., Rastall R.A., Roberfroid M.B. (2004). Dietary modulation of the human colonic microbiota: Updating the concept of prebiotics. Nutr. Res. Rev..

[5] Roberfroid M., Gibson G.R., Hoyles L., McCartney A.L., Rastall R., Rowland I., Wolvers D., Watzl B., Szajewska H., Stahl B. (2010). Prebiotic effects: Metabolic and health benefits. Br. J. Nutr..

[6] Collins S., Reid G. (2016). Distant Site Effects of Ingested Prebiotics. Nutrients.

[7] Salminen S., Bouley C., Boutron-Ruault M.C., Cummings J.H., Franck A., Gibson G.R., Isolauri E., Moreau M.C., Roberfroid M., Rowland I. (1998). Functional food science and gastrointestinal physiology and function. Br. J. Nutr..

[8] Skórka A., Pieścik-Lech M., Kołodziej M., Szajewska H. (2018). Infant formulae supplemented with prebiotics: Are they better than unsupplemented formulae? An updated systematic review. Br. J. Nutr..

[9] Gibson G.R., Roberfroid M.B. (1995). Dietary modulation of the human colonic microbiota: Introducing the concept of prebiotics. J. Nutr..

[10] Gibson G.R., Hutkins R., Sanders M.E., Prescott S.L., Reimer R.A., Salminen S.J., Scott K., Stanton C., Swanson K.S., Cani P.D. (2017). Expert consensus document: The International Scientific Association for Probiotics and Prebiotics (ISAPP) consensus statement on the definition and scope of prebiotics. Nat. Rev. Gastroenterol. Hepatol..

[11] Closa-Monasterolo R., Gispert-Llaurado M., Luque V., Ferre N., Rubio-Torrents C., Zaragoza-Jordana M., Escribano J. (2013). Safety and efficacy of inulin and oligofructose supplementation in infant formula: Results from a randomized clinical trial. Clin. Nutr..

[12] Veereman-Wauters G., Staelens S., van de Broek H., Plaskie K., Wesling F., Roger L.C., McCartney A.L., Assam P. (2011). Physiological and bifidogenic effects of prebiotic supplements in infant formulae. J. Pediatr. Gastroenterol. Nutr..

[13] Waligora-Dupriet A.J., Campeotto F., Nicolis I., Bonet A., Soulaines P., Dupont C., Butel M.J. (2007). Effect of oligofructose supplementation on gut microflora and well-being in young children attending a day care centre. Int. J. Food Microbiol..

[14] Lohner S., Jakobik V., Mihályi K., Soldi S., Vasileiadis S., Theis S., Sailer M., Sieland C., Berényi K., Boehm G. (2018). Inulin-Type Fructan Supplementation of 3- to 6-Year-Old Children Is Associated with Higher Fecal Bifidobacterium Concentrations and Fewer Febrile Episodes Requiring Medical Attention. J. Nutr..

[15] Lohner S., Küllenberg D., Antes G., Decsi T., Meerpohl J.J. (2014). Prebiotics in healthy infants and children for prevention of acute infectious diseases: A systematic review and meta-analysis. Nutr. Rev..

[16] Arslanoglu S., Moro G.E., Boehm G. (2007). Early supplementation of prebiotic oligosaccharides protects formula-fed infants against infections during the first 6 months of life. J. Nutr..

[17] Puccio G., Alliet P., Cajozzo C., Janssens E., Corsello G., Sprenger N., Wernimont S., Egli D., Gosoniu L., Steenhout P. (2017). Effects of Infant Formula with Human Milk Oligosaccharides on Growth and Morbidity: A Randomized Multicenter Trial. J. Pediatr. Gastroenterol. Nutr..

[18] Saavedra J.M., Tschernia A. (2002). Human studies with probiotics and prebiotics: Clinical implications. Br. J. Nutr..

[19] Watzl B., Girrbach S., Roller M. (2005). Inulin, oligofructose and immunomodulation. Br. J. Nutr..

[20] Sokol H., Seksik P., Furet J.P., Firmesse O., Nion-Larmurier I., Beaugerie L., Cosnes J., Corthier G., Marteau P., Doré J. (2009). Low counts of Faecalibacterium prausnitzii in colitis microbiota. Inflamm. Bowel Dis..

[21] Matsuki T., Watanabe K., Fujimoto J., Kado Y., Takada T., Matsumoto K., Tanaka R. (2004). Quantitative PCR with 16S rRNA-gene-targeted species-specific primers for analysis of human intestinal bifidobacteria. Appl. Environ. Microbiol..

[22] Bartosch S., Fite A., Macfarlane G.T., McMurdo M.E. (2004). Characterization of bacterial communities in feces from healthy elderly volunteers and hospitalized elderly patients by using real-time PCR and effects of antibiotic treatment on the fecal microbiota. Appl. Environ. Microbiol..

[23] Weizman Z., Asli G., Alsheikh A. (2005). Effect of a probiotic infant formula on infections in child care centers: Comparison of two probiotic agents. Pediatrics.

[24] Euler A.R., Mitchell D.K., Kline R., Pickering L.K. (2005). Prebiotic effect of fructo-oligosaccharide supplemented term infant formula at two concentrations compared with unsupplemented formula and human milk. J. Pediatr. Gastroenterol. Nutr..

[25] Brunser O., Figueroa G., Gotteland M., Haschke-Becher E., Magliola C., Rochat F., Cruchet S., Palframan R., Gibson G., Chauffard F. (2006). Effects of probiotic or prebiotic supplemented milk formulas on fecal microbiota composition of infants. Asia Pac. J. Clin. Nutr..

[26] Yao M., Lien E.L., Capeding M.R., Fitzgerald M., Ramanujam K., Yuhas R., Northington R., Lebumfacil J., Wang L., DeRusso P.A. (2014). Effects of term infant formulas containing high sn-2 palmitate with and without oligofructose on stool composition, stool characteristics, and bifidogenicity. J. Pediatr. Gastroenterol. Nutr..

[27] Wernimont S., Northington R., Kullen M.J., Yao M., Bettler J. (2015). Effect of an α-lactalbumin-enriched infant formula supplemented with oligofructose on fecal microbiota, stool characteristics, and hydration status: A randomized, double-blind, controlled trial. Clin. Pediatr. Phila.

[28] Scholtens P.A., Alliet P., Raes M., Alles M.S., Kroes H., Boehm G., Knippels L.M., Knol J., Vandenplas Y. (2008). Fecal secretory immunoglobulin A is increased in healthy infants who receive a formula with short-chain galacto-oligosaccharides and long-chain fructo-oligosaccharides. J. Nutr..

[29] Bakker-Zierikzee A.M., Tol E.A., Kroes H., Alles M.S., Kok F.J., Bindels J.G. (2006). Faecal SIgA secretion in infants fed on pre- or probiotic infant formula. Pediatr. Allergy Immunol..

[30] Moro G., Arslanoglu S., Stahl B., Jelinek J., Wahn U., Boehm G. (2006). A mixture of prebiotic oligosaccharides reduces the incidence of atopic dermatitis during the first six months of age. Arch. Dis. Child..

[31] Arslanoglu S., Moro G.E., Schmitt J., Tandoi L., Rizzardi S., Boehm G. (2008). Early dietary intervention with a mixture of prebiotic oligosaccharides reduces the incidence of allergic manifestations and infections during the first two years of life. J. Nutr..

[32] Boehm G., Fanaro S., Moro G., Knol J., Arslanoglu S., Mosca F., Stahl B. (2004). Prebiotic oligosaccharides in infant nutrition: Effects on intestinal flora. Agro Food Ind. Hi Tech.

[33] Oozeer R., van Limpt K., Ludwig T., Ben Amor K., Martin R., Wind R.D., Boehm G., Knol J. (2013). Intestinal microbiology in early life: Specific prebiotics can have similar functionalities as human-milk oligosaccharides. Am. J. Clin. Nutr..

[34] Vandenplas Y., Veereman-Wauters G., Greef E.D., Peeters S., Casteels A., Mahler T., Devreker T., Hauser B. (2011). Probiotics and prebiotics in prevention and treatment of diseases in infants and children. J. Pediatr. Rio J..

[35] Bettler J., Euler A.R. (2006). An evaluation of the growth of term infants fed formula supplemented with fructo-oligosaccharide. Int. J. Probiotics Prebiotics.

[36] Scholtens P.A., Goossens D.A., Staiano A. (2014). Stool characteristics of infants receiving short-chain galacto-oligosaccharides and long-chain fructo-oligosaccharides: A review. World J. Gastroenterol..

