# Supplementary material for: Long-Term Safety and Efficacy of Prebiotic Enriched Infant Formula—A Randomized Controlled Trial
Source: Nutrients. 2021 Apr 13;13(4):1276. doi: 10.3390/nu13041276 (PMC8070502; doi:10.3390/nu13041276)
Supplement: Supplementary file 1 [file nutrients-13-01276-s001.pdf]

Long-term safety and efficacy of prebiotic enriched infant formula– A Randomized Controlled Trial

Franka Neumer [1,*], Orenci Urraca [2], Joaquin Alonso [3], Jesús Palencia [4], Vicente Varea [5], Stephan Theis [1], Maria Rodriguez-Palmero [6], José Antonio Moreno-Muñoz [6], Francisco Guarner [7], Gigi Veereman [8], Yvan Vandenplas [8], Cristina Campoy [9]

Supplemental Tables

Supplemental Table S1: Study formula characteristics.

		Infant Formula		Follow-on formula	
		**Control**	**Experimental**	**Control**	**Experimental**
		Per 100 ml			
**Energy value**	kcal (kJ)	67 (280)	66 (276)	69 (289)	68 (285)
**Proteins**	g	1.4	1.4	1.8	1.8
**Fat**	g	3.5	3.5	3.2	3.2
**Carbohydrates**	g	7.5	6.8	8.3	7.6
**Lactose**	g	6.1	6.1	6.2	6.2
**Synergy1**	g	0	0.8	0	0.8
**Choline**	mg	7.1	7.1	7.3	7.3
**Taurine**	mg	4.5	4.5	4.8	4.8
**Inositol**	mg	3.5	3.5	3.6	3.6
**L-carnitine**	mg	2.4	2.4	2.3	2.3
**Minerals**	g	0.4	0.4	0.6	0.6
Sodium	mg	25	25	42	42
Potassium	mg	75	75	113	113
Chloride	mg	41	41	81	81

Calcium	mg	59	59	99.2	99.2
Phosphorus	mg	32	32	55	55
Iron	mg	0.8	0.8	1.2	1.2
Magnesium	mg	5.9	5.9	8.7	8.7
Zinc	mg	0.6	0.6	0.7	0.7
Copper	mcg	42	42	65	65
Iodine	mcg	9.8	9.8	12	12
Manganese	mcg	7	7	11.6	11.6
Selenium	mcg	2.1	2.1	2.1	2.1
**Vitamins**					
Vitamin A	mcg	90	90	90	90
	IU	299	299	300	300
Vitamin D	mcg	1.44	1.44	1.7	1.7
	IU	58	58	70	70
Vitamin E	mg	3.5	3.5	3.5	3.5
Vitamin K	mcg	5.9	5.9	6.1	6.1
Vitamin C	mg	8.4	8.4	7.3	7.3
Thiamin (B1)	mcg	73	73	80	80
Riboflavin (B2)	mcg	87	87	128	128
Nicotinamide (B3)	mg	0.8	0.8	1.5	1.5
Pantothenic acid	mg	0.5	0.5	0.5	0.5
Vitamin B6	mcg	116	116	119	119
Folic acid	mcg	8.4	8.4	15	15
Vitamin B12	mcg	0.3	0.3	0.3	0.3
Biotin	mcg	2.2	2.2	2.3	2.3

Supplemental Table S2 Primer selection for qPCR.

Target	Primer name	Sequence 5´-3´	Annealing	Reference
Total bacteria	F_Bakt1369<br>R_Prok1492	CGG TGA ATA CGT TCC CGG<br>TAC GGC TAC CTT GTT ACG ACT T	60 °C	Sokol et al. 2009[17]
Clostridium leptum subgroup (clostridial cluster IV)	Sg-Clept-F<br>Sg-Clept-R3	GCA CAA GCA GTG GAG T<br>CTT CCT CCG TTT TGT CAA	50 °C	Matsuki et al. 2004[18]
Clostridium coccoides group (clostridial cluster XIVa)	Ccoc 07<br>Ccoc 14	GAC GCC GCG TGA AGG A<br>AGC CCC AGC CTT TCA CAT C	60 °C	Sokol et al. 2009[17]
Bacteroides	Bacter 11<br>Bacter 08	CCT WCG ATG GAT AGG GGT T<br>CAC GCT ACT TGG CTG GTT CAG	60 °C	Sokol et al. 2009[17]
Bifidobacterium	F_Bifid09c<br>R_Bifid06	CGG GTG AGT AAT GCG TGA CC<br>TGA TAG GAC GCG ACC CCA	60 °C	Sokol et al. 2009[17]
Enterobacteriaceae	ECO1457F<br>ECO1652R	CAT TGA CGT TAC CCG CAG AAG AAG C<br>CTC TAC GAG ACT CAA GCT TGC	60°C	Bartosch et al. 2004[19]

Supplemental Table S3 Growth related variables of infants following consumption of either prebiotic vs. control formula at following time-points: age of 2, 4, 6, 9 and 12 months (M2-M12, respectively).

	M2		M4		M6		M9		M12	
	**Prebiotic**	**Control**	**Prebiotic**	**Control**	**Prebiotic**	**Control**	**Prebiotic**	**Control**	**Prebiotic**	**Control**
**Age (days)**	66.61 (22.29)	69.97 (23.33)	126.58 (14.50)	128.51 (25.66)	184.90 (10.07)	183.00 (19.24)	278.30 (15.43)	277.0 (16.59)	369.87 (17.12)	368.87 (14.57)
**Weight (g)**	5468 (660)	5382 (806)	6841 (754)	7060 (762)	8004 (869)	8152 (884)	9175 (963)	9455 (934)	10137 (1136)	10408 (1065)
**Length (cm)**	58.02 (2.30)	57.31 (3.24)	63.27 (3.31)	63.55 (4.29)	67.29 (2.98)	67.36 (3.41)	72.12 (2.68)	72.45 (5.10)	75.42 (4.89)	76.61 (3.29)
**BMI (kg/m^2)**	16.22 (1.52)	16.32 (1.24)	17.10 (1.58)	17.57 (2.20)	17.67 (1.87)	18.00 (1.87)	17.63 (1.60)	18.28 (3.71)	18.14 (5.21)	17.73 (1.36)
**HC (cm)**	39.29 (1.49)	39.63 (1.64)	41.56 (1.72)	42.28 (1.81)	43.25 (1.53)	44.63 (1.73)	44.76 (1.77)	47.07 (1.91)	47.62 (1.73)	46.65 (1.74)

Data are presented as means and SD (standard deviation, in brackets). BMI: Body mass index, HC: Head circumference.

Supplemental Table S4 Amount of formula ingested following consumption of either prebiotic vs. control formula at following time-points: age of 2, 4, 6, 9 and 12 months (M2-M12, respectively).

	M2		M4		M6		M9		M12	
	**Prebiotic**	**Control**	**Prebiotic**	**Control**	**Prebiotic**	**Control**	**Prebiotic**	**Control**	**Prebiotic**	**Control**
**Amount of formula ingested (ml/day)**	770.4 (125.1)	816.0 (142.0)	809.9 (180.1)	885.4 (159.4)*	721.3 (226.5)	764.0 (233.3)	595.7 (203.6)	648.5 (255.9)	568 (201.5)	638.5 (246.8)

Data are presented as means and SD (standard deviation, in brackets).

*Statistical differences between the groups (p<0.05)

Supplemental Table S5 Number of infections until 12 months of age.

		NUMBER OF INFECTIONS		
		**N**	**Mean**	**SD**
**VISIT (MONTH)**	**GROUP**			
**2**	**Control**	33	0.00	0.00
	**Prebiotic**	26	0.00	0.00
**4**	**Control**	59	0.32	0.63
	**Prebiotic**	52	0.23	0.65
**6**	**Control**	69	0.32	0.80
	**Prebiotic**	71	0.37	0.85
**9**	**Control**	66	0.58	1.15
	**Prebiotic**	65	0.68	1.24
**12**	**Control**	62	0.55	1.04
	**Prebiotic**	61	0.64	1.05

Supplemental Table S6 Digestive symptoms of the infants by feeding group and study time.

Visit month	VOMITS		REGURGITATIONS		FLATULENCE	
	**Prebiotic**	**Control**	**Prebiotic**	**Control**	**Prebiotic**	**Control**
	Mean (SD)		*Mean (SD)*		%	
**2**	0.32 (0.62)	0.37 (0.77)	1.99 (1.58)	2.12 (1.67)	58.8	47.6
**4**	0.28 (0.33)	0.90 (1.29)	1.99 (1.92)	1.49 (1.80)	31.2	34.4
**6**	0.23 (0.25)	0.36 (0.46)	1.26 (1.29)	1.11 (1.47)	29.7	20.5
**9**	0.02 (0.04)	0.03 (0.05)	0.21 (0.42)	0.36 (0.92)	26.9	9.37
**12**	0.01 (0.04)	0.01 (0.02)	0.06 (0.32)	0.09 (0.26)	21.7	12

For vomits and regurgitation, results are expressed as number of events per day. For flatulence, percentage of infants with flatulence is presented.

*Statistical differences between the groups ($p<0.05$)
